# Unusual Adolescent Abdominal Tumors Presenting With Neuropsychiatric and Massive Abdominopelvic Manifestations: A Report of Two Cases

**DOI:** 10.7759/cureus.78899

**Published:** 2025-02-12

**Authors:** Avinash Hiremath, Mohammed Alblooshi, Mamoun AlMarzouqi, Diary Mohammed, Pawan Kashyape

**Affiliations:** 1 Pediatric Surgery, Al Jalila Children's Specialty Hospital, Dubai, ARE; 2 Pediatric Surgery, Tawam Hospital, Al Ain, ARE; 3 Pediatric Neurology, Al Jalila Children's Specialty Hospital, Dubai, ARE

**Keywords:** adolescent abdominal tumors, nmdar encephalitis, ovarian teratoma, pediatric surgical management, uterine leiomyoma

## Abstract

Abdominal tumors in adolescence represent a diverse group of pathologies that can present atypically, ranging from neuropsychiatric disturbances to significant abdominal distension. The objective of this study is to report two rare cases that highlight the diagnostic and therapeutic challenges of abdominal tumors in this age group. In the first case, a 12-year-old girl presented with a 10-day history of acute-onset neuropsychiatric symptoms, including hallucinations and cognitive decline, leading to a diagnosis of anti-N-methyl-D-aspartate receptor (NMDAR) encephalitis. Although the initial ultrasound was non-diagnostic, subsequent magnetic resonance imaging (MRI) revealed a 3-cm ovarian dermoid cyst. Prompt surgical resection combined with immunotherapy resulted in rapid neurological improvement, with complete recovery observed within days and sustained at a one-year follow-up. In the second case, a 14-year-old girl exhibited progressive abdominal distension over one month and was found to have a large, predominantly solid pelvic mass. Detailed imaging studies, including computed tomography (CT), delineated a bilobed mass measuring up to 30 cm in maximum dimension. Intraoperative findings confirmed the mass to be an intramural uterine leiomyoma; surgical excision led to the resolution of symptoms and normalization of laboratory parameters (hemoglobin improved from 8.6 g/dL), with no recurrence noted during follow-up. These cases quantitatively underscore that even benign tumors measuring 3 cm and 30 cm, respectively, lead to significant morbidity. Ultimately, our findings emphasize the importance of a high index of suspicion, repeated high-resolution imaging, and a multidisciplinary approach to ensure timely diagnosis and optimal management, thereby contributing to improved clinical strategies in the management of atypical abdominal tumors in adolescents.

## Introduction

Abdominal tumors in adolescents, though comparatively rare, can present with a wide spectrum of signs and symptoms, posing significant challenges in diagnosis and management [1]. Their clinical manifestations are often nonspecific, ranging from subtle abdominal distension or systemic pain and even neuropsychiatric symptoms. Despite advances in imaging and clinical assessment, a notable research gap remains in the systematic characterization and management of atypically presenting abdominal tumors in this age group, particularly those associated with paraneoplastic or autoimmune phenomena. Such diverse presentations may stem from the tumor’s biological behavior or, in certain circumstances, from paraneoplastic or autoimmune phenomena [2]. Consequently, these atypical presentations may obscure the underlying pathology, resulting in delayed or missed diagnoses.

Among the notable abdominal tumors in this age group, ovarian teratomas hold particular clinical significance due to their association with autoimmune encephalitides, most prominently anti-N-methyl-D-aspartate receptor (NMDAR) encephalitis. Anti-NMDAR encephalitis is characterized by autoantibodies targeting neuronal NMDA receptors, leading to a constellation of neuropsychiatric manifestations, including behavioral changes, memory deficits, seizures, and movement disorders [3]. Ovarian teratomas, which frequently harbor ectopic neural tissue, are identified as the triggering lesion in a substantial proportion of these cases, although the exact incidence in pediatric populations remains less well-defined [4,5]. This lack of precise epidemiological data and a standardized diagnostic workup for ovarian teratomas associated with neuropsychiatric symptoms in adolescents represents a critical gap in the current literature. Prompt recognition and early multimodal intervention, immunotherapy combined with surgical resection, can significantly improve outcomes [6].

Conversely, uterine leiomyomas (fibroids), the most common benign pelvic tumors in adult women, are exceedingly rare in the pediatric population, with an incidence of less than 1% in adolescents [1]. When they do occur, they can reach substantial size and create mass effects, resulting in abdominal distension, pain, and compression of neighboring structures. Because gynecological malignancies remain a prime concern in any adolescent presenting with a pelvic or abdominal mass, uterine fibroids are often not the first consideration [7]. Although these tumors may present with cyclical or heavy menstrual bleeding in adult women, adolescents might experience less typical or even silent symptomatology. Imaging modalities such as ultrasonography, computed tomography (CT), and magnetic resonance imaging (MRI) are instrumental in delineating the lesion’s origin, yet definitive diagnosis often relies on surgical exploration and histopathological assessment [8,9]. Moreover, the literature lacks comprehensive case analyses that focus on the diagnostic challenges and management strategies for giant uterine leiomyomas in this age group.

Given the rarity and potential for atypical presentations, abdominal tumors in adolescents warrant a high index of suspicion and a methodical diagnostic approach. Early detection is crucial for determining appropriate management, ranging from immunotherapy and surgical resection in teratoma-associated encephalitis to fertility-sparing myomectomy in the case of uterine leiomyomas [10]. Herein, we describe two unusual presentations of abdominal tumors in adolescent females: one presenting with autoimmune encephalitis secondary to ovarian teratoma and another manifesting as a large uterine leiomyoma. Through these cases, we underscore the need for a broad differential diagnosis, timely imaging, and multidisciplinary intervention to optimize outcomes and preserve function.

To address these gaps, the primary objective of this study is to present and analyze two unusual cases of abdominal tumors in adolescent females, detailing their clinical presentations, diagnostic workups, and therapeutic interventions. Specifically, we aim to illustrate the diagnostic challenges posed by ovarian teratoma-associated anti-NMDAR encephalitis and the management complexities of a giant uterine leiomyoma. We employ a case report design that retrospectively reviews clinical data, imaging findings, surgical details, and follow-up outcomes to provide a comprehensive account of these rare presentations. Through these cases, we underscore the necessity for a high index of suspicion, repeated high-resolution imaging, and a multidisciplinary approach to ensure timely diagnosis and optimal management, thereby contributing valuable insights into the management of atypical abdominal tumors in adolescents.

## Case presentation

Case 1

A 12-year-old previously healthy girl presented with a 10-day history of progressively worsening behavioral changes, including paranoia, visual hallucinations, and cognitive decline. Notably, her past medical, psychiatric, and family histories were unremarkable. On initial evaluation, she was afebrile with normal vital signs; however, she demonstrated fluctuating levels of consciousness, marked agitation, and occasional speech arrest.

Day 1: Presentation and Initial Evaluation

Initial laboratory evaluations, including a complete blood count and basic metabolic panel, were within normal limits, and a toxicology screen was negative. Given the prominent neuropsychiatric symptoms, autoimmune encephalitis was promptly considered in the differential diagnosis.

Day 2: Diagnostic Workup

**Figure 1:**
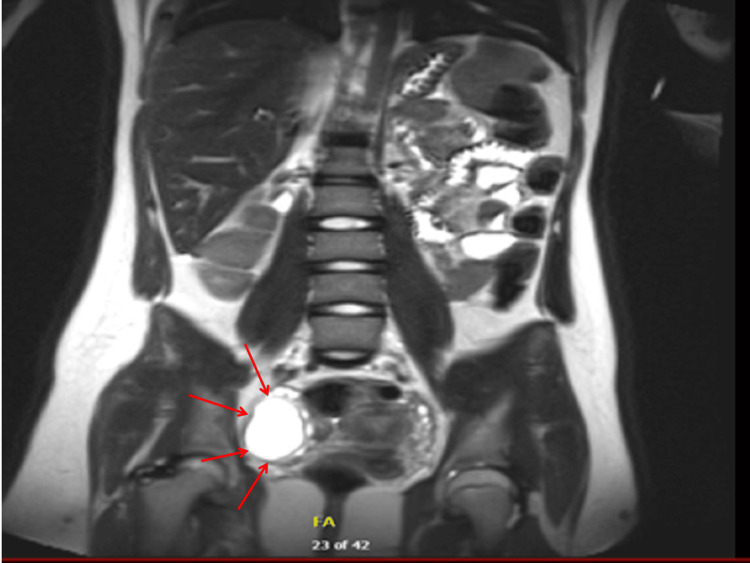
Right ovarian tumor MRI coronal image (Case 1) Coronal T2-weighted MRI showing a well-defined, approximately 3-cm right ovarian cystic lesion. The lesion exhibits both fluid and soft-tissue components consistent with a mature cystic teratoma (dermoid cyst), located lateral to the uterus and adjacent to the pelvic brim.

**Figure 2:**
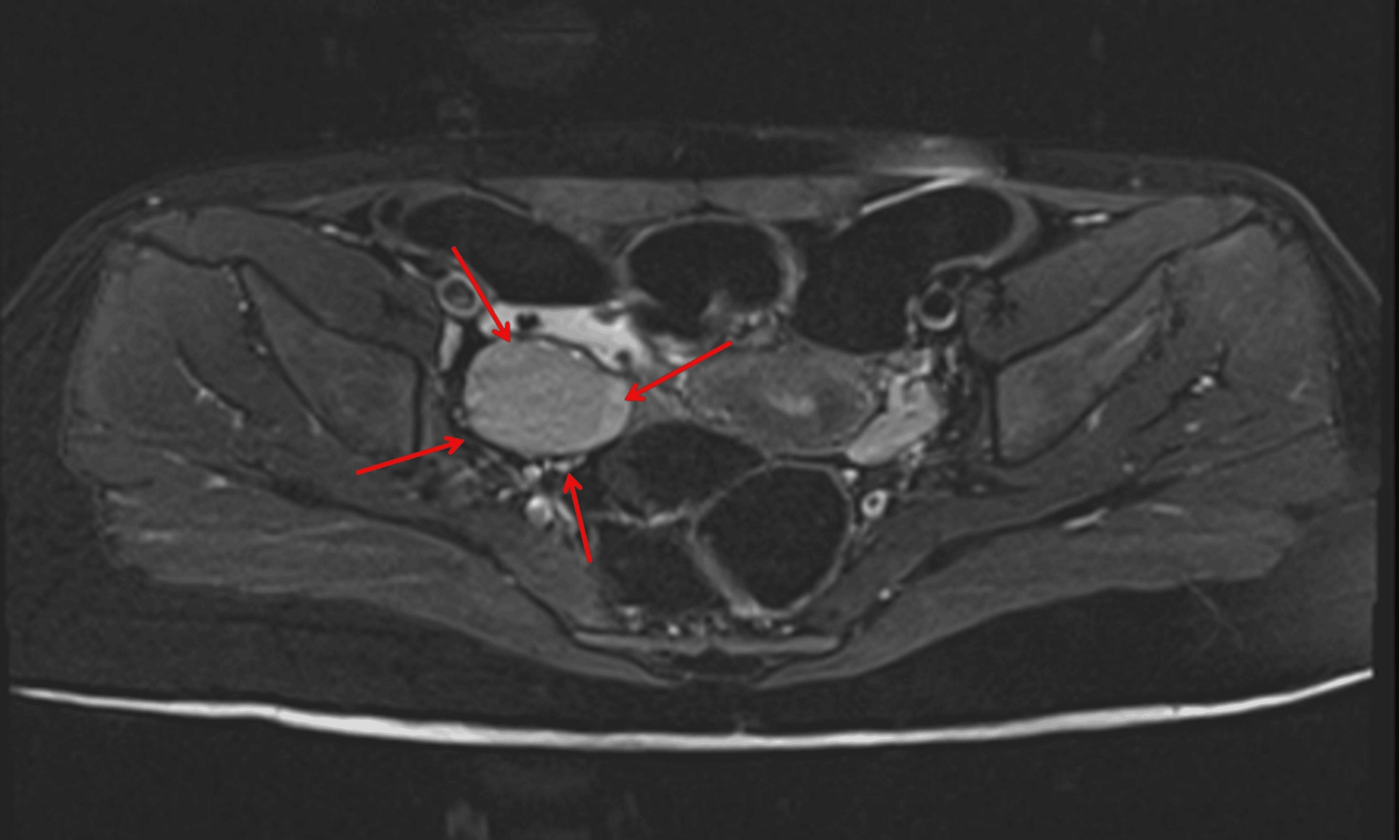
Right ovarian tumor MRI transverse image (Case 1) Transverse T2-weighted MRI showing a well-defined, approximately 3-cm right ovarian cystic lesion. Both fluid and soft-tissue components are visible, consistent with a mature cystic teratoma (dermoid cyst). The lesion is situated lateral to the uterus, reinforcing its ovarian origin.

A lumbar puncture was performed, which revealed lymphocytic pleocytosis and elevated protein levels. Based on the clinical suspicion of anti-N-methyl-D-aspartate receptor (NMDAR) encephalitis, empirical treatment with high-dose intravenous methylprednisolone followed by intravenous immunoglobulins was initiated. While awaiting confirmatory antibody testing, abdominal imaging was undertaken to rule out an occult neoplasm. Although the initial pelvic ultrasound was noncontributory, a subsequent MRI of the abdomen and pelvis disclosed a cystic lesion in the right ovary with a small solid component, highly suggestive of an ovarian teratoma (Figure 1: coronal view; Figure 2: transverse view). Tumor markers, including α-fetoprotein and β-human chorionic gonadotropin, were unremarkable.

Day 5: Intervention

Following definitive confirmation of anti-NMDAR encephalitis via CSF autoantibody testing, the patient underwent a laparoscopic right ovarian cystectomy. Intraoperatively, a 3 cm mature cystic teratoma was identified and completely resected. Histopathological examination confirmed the presence of skin appendages and hair shafts, consistent with a benign dermoid cyst.

Postoperative Course and Follow-Up

Postoperatively, the patient exhibited marked neurological improvement within days. At one-year follow-up, she had returned to age-appropriate functioning in both school and home settings, with no evidence of disease recurrence.

Key Learning Points

When adolescents present with acute neuropsychiatric symptoms, clinicians should consider the possibility of autoimmune encephalitis, particularly anti-NMDAR encephalitis, which necessitates a thorough evaluation of an underlying ovarian teratoma. Moreover, even if an initial ultrasound does not yield conclusive results, this does not rule out the presence of a teratoma; indeed, high-resolution imaging such as MRI can be crucial in identifying small lesions. Consequently, the combination of prompt immunotherapy and surgical resection can lead to rapid and sustained neurological recovery.

Case 2

A 14-year-old girl presented to the emergency department with a month-long history of progressive abdominal distension and a one-day history of dull, localized lower abdominal pain. She denied any significant weight loss, vomiting, or changes in bowel habits. Menarche was at age 12, with regular monthly cycles and no abnormal uterine bleeding. On examination, her vital signs were stable, and a firm, nontender mass was palpable from the hypogastrium extending to the epigastric region. Laboratory tests revealed a hemoglobin of 8.6 g/dL with normal renal and liver function tests. The reduced hemoglobin raised concerns regarding possible chronic blood loss or a mass effect from a large intra-abdominal lesion.

**Figure 3:**
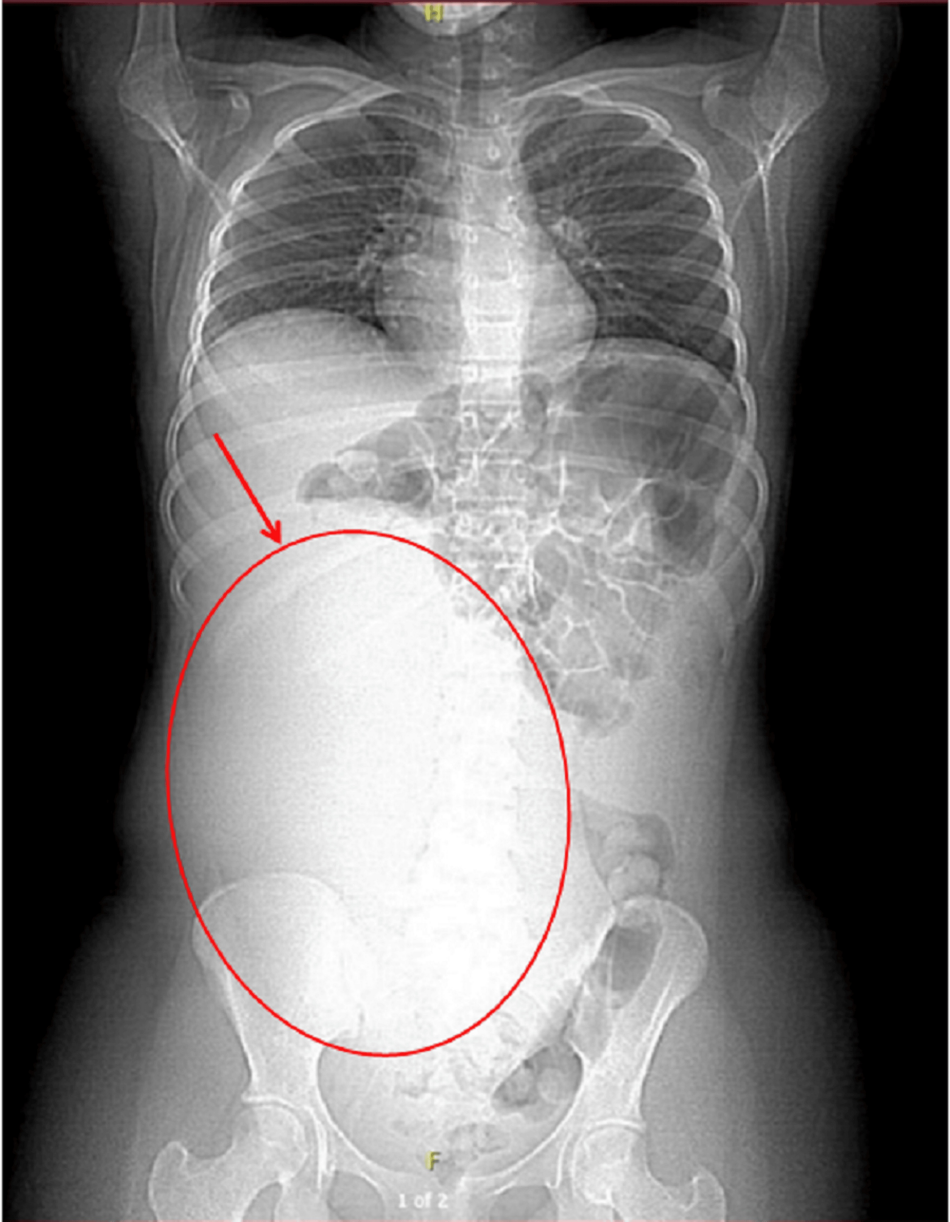
Upright abdominal X-ray showing mass displacement (Case 2)

**Figure 4:**
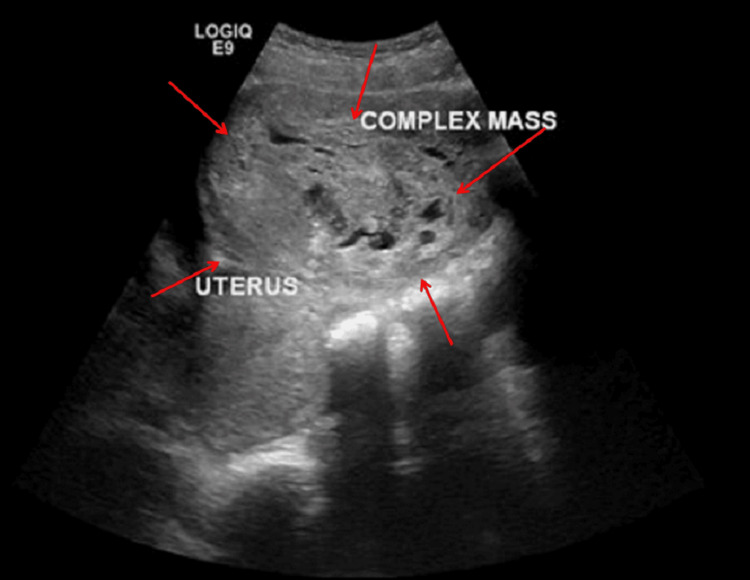
Ultrasound image of uterine mass adjacent to the uterus (Case 2)

**Figure 5:**
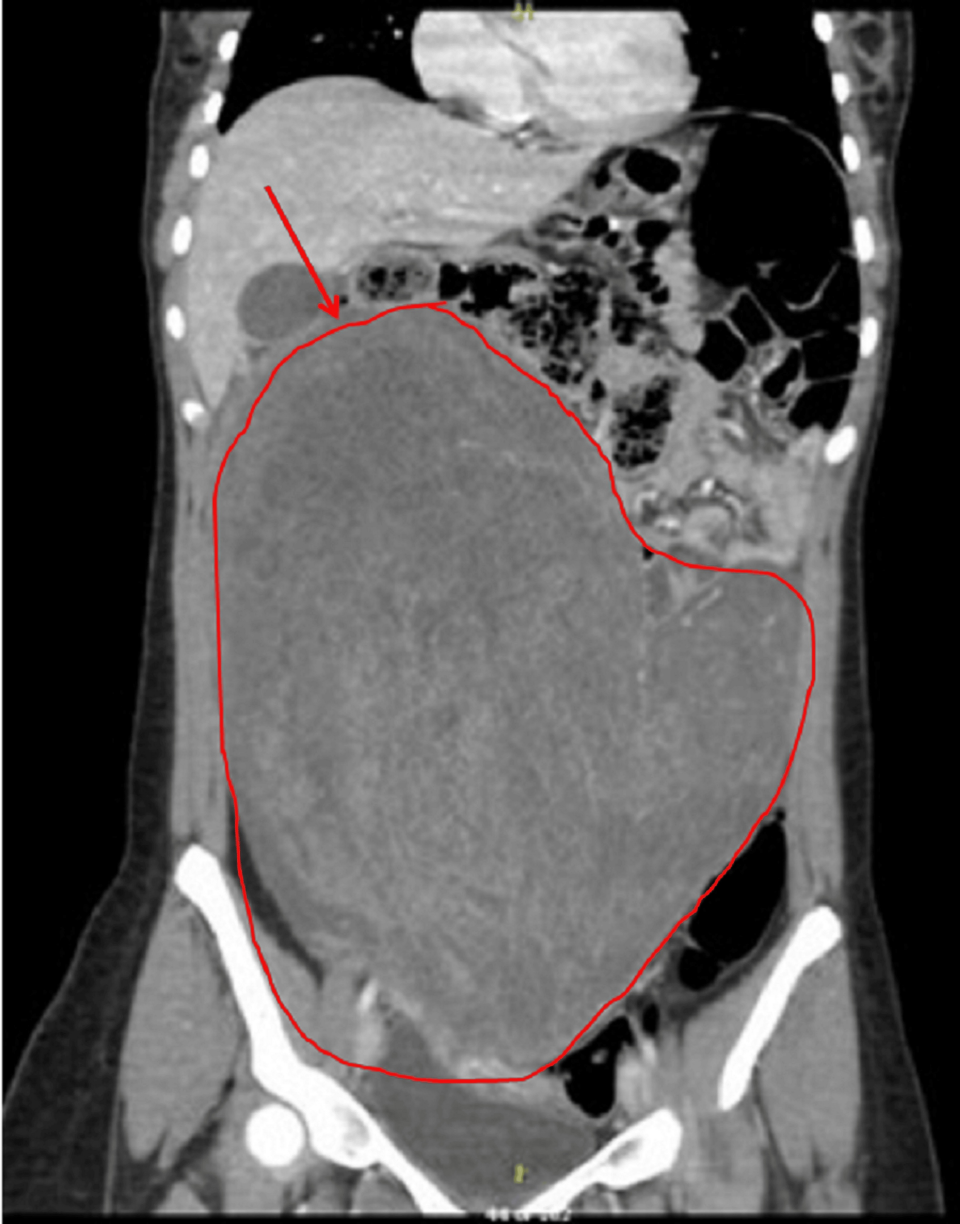
Coronal CT scan showing the mass compressing bladder and bowel (Case 2)

**Figure 6:**
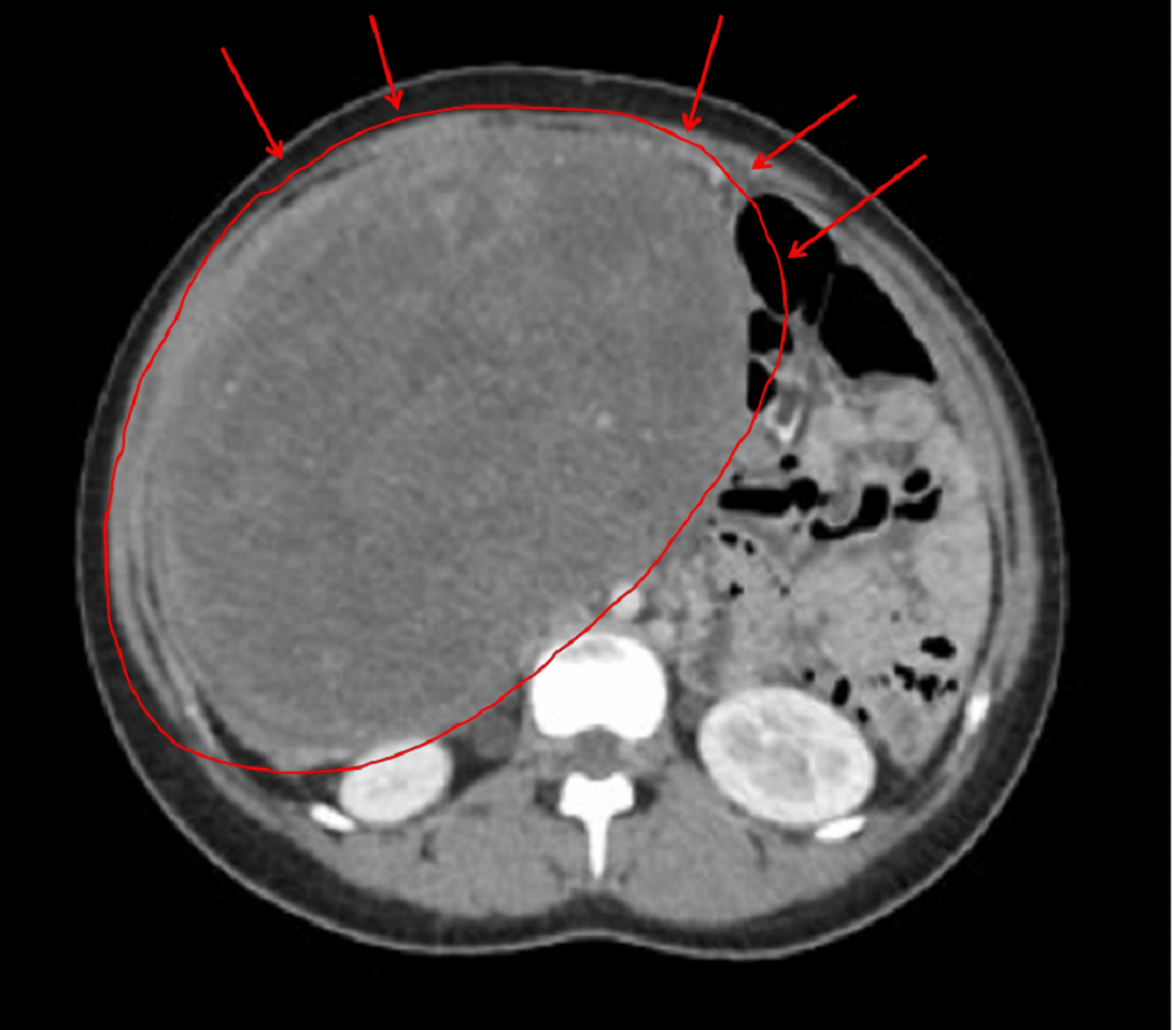
Axial CT scan showing the central location and enhancement of the mass (Case 2)

An upright abdominal X-ray demonstrated a large soft tissue density displacing bowel loops to the left upper quadrant, without signs of obstruction or free air (Figure 3). Pelvic ultrasound showed a sizable complex solid lesion, measuring approximately 22 × 18 cm, contiguous with the uterus (Figure 4). The right ovary appeared normal, whereas the left ovary could not be visualized. These imaging findings prompted consideration of a broad differential diagnosis, including ovarian germ cell tumors, uterine sarcoma, and benign uterine pathologies such as leiomyoma. Subsequent contrast-enhanced computed tomography (CT) of the abdomen and pelvis revealed a bilobed, predominantly solid mass with areas of necrosis; it exerted a mass effect on the bladder and inferior vena cava (Figure 5, coronal view; Figure 6, axial view). Tumor markers, including α-fetoprotein, β-human chorionic gonadotropin, and CA-125, were within normal limits or only mildly elevated, which reduced the likelihood of a malignant process.

**Figure 7:**
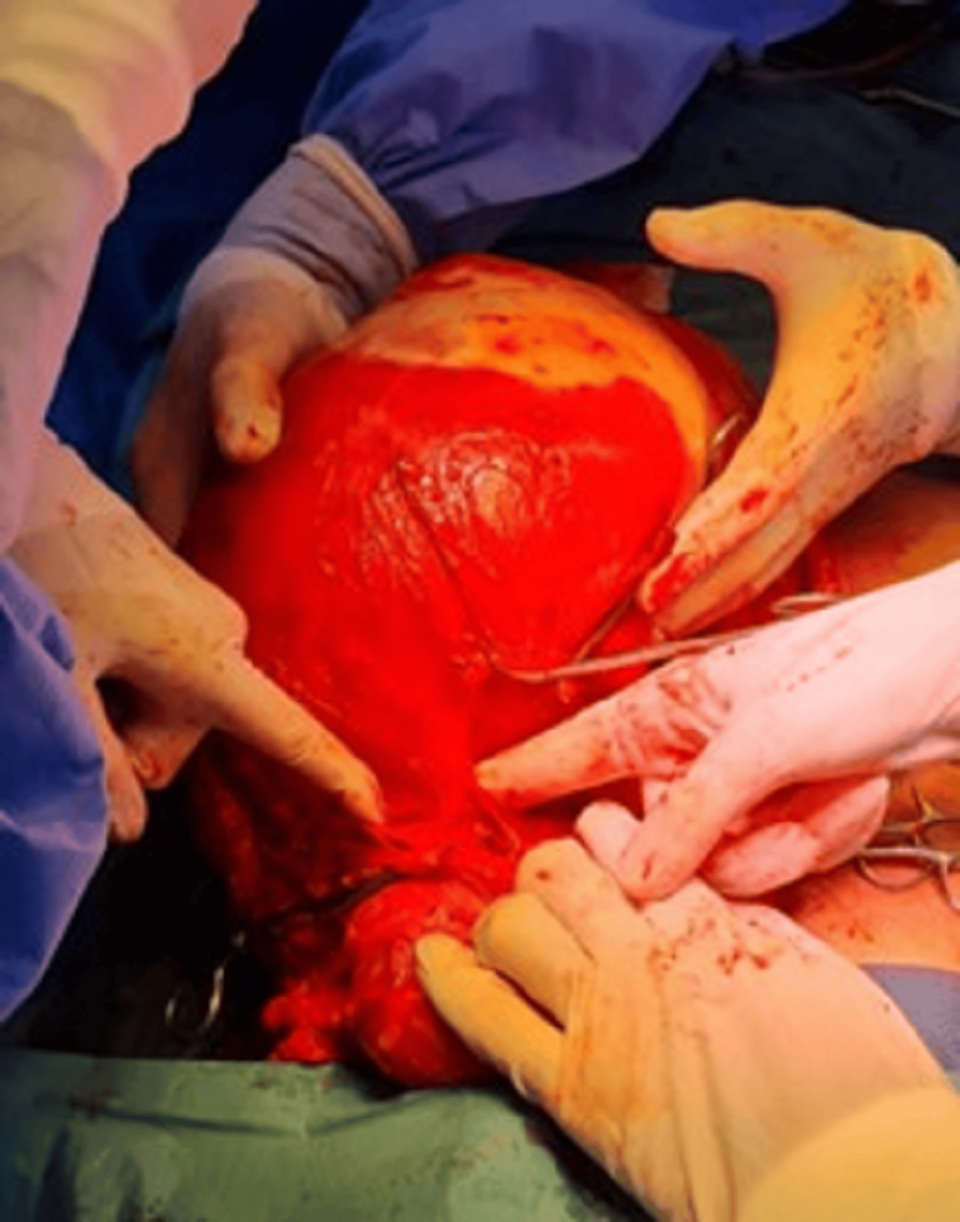
Intraoperative image showing initial exposure of the large uterine mass (Case 2)

**Figure 8:**
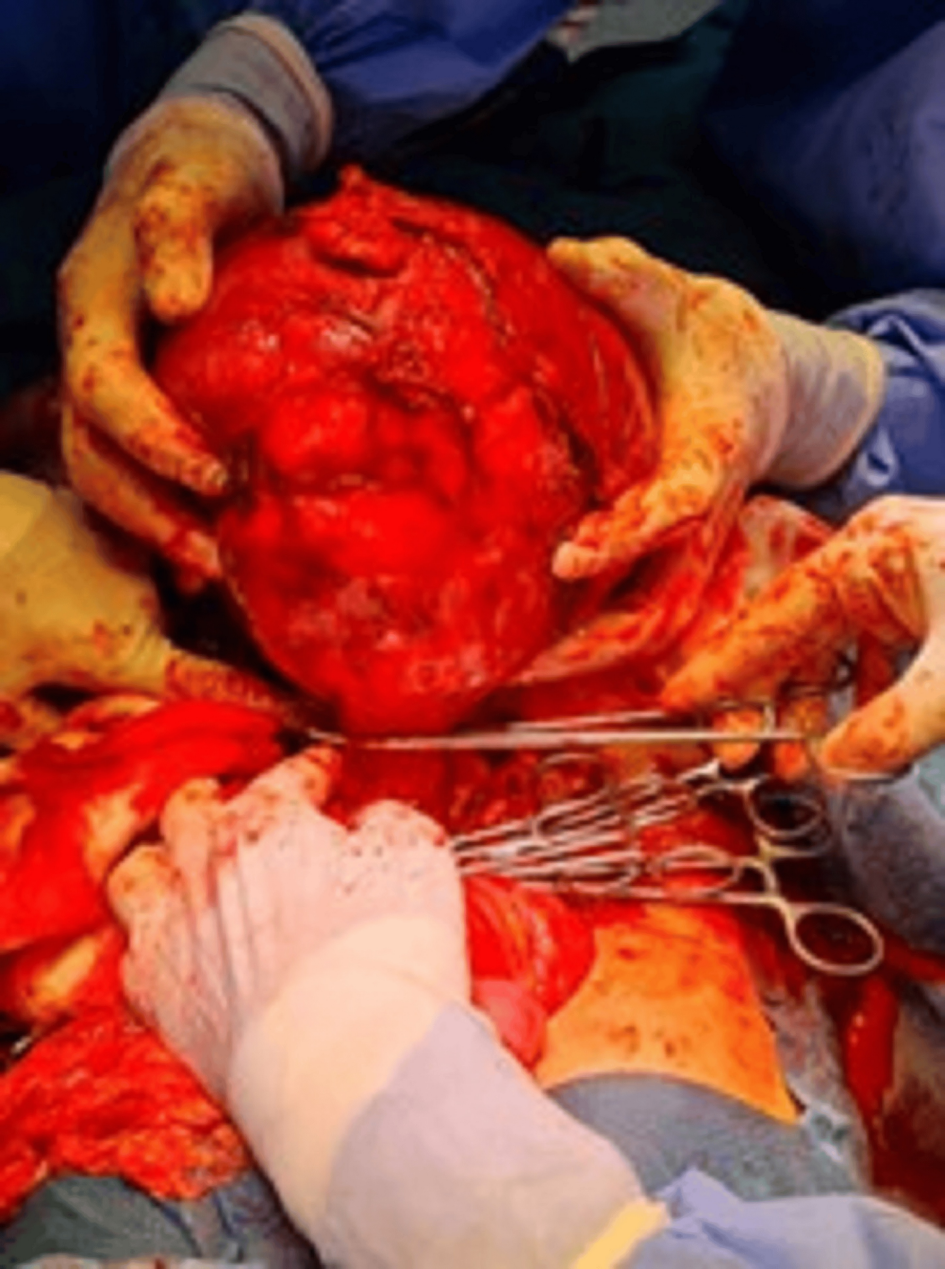
Intraoperative image showing the dissection of the mass from its attachments (Case 2)

**Figure 9:**
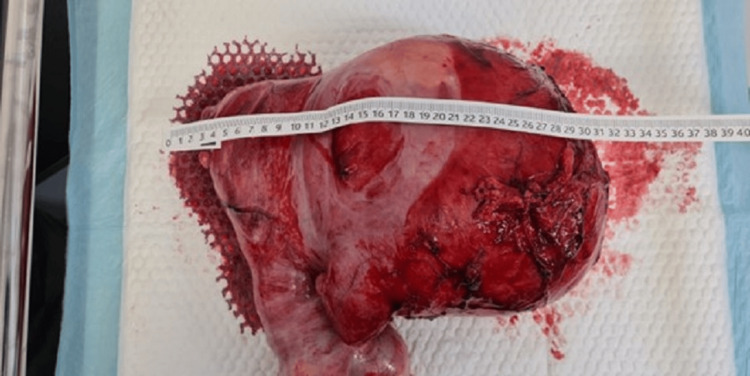
Measurement of the excised 30 cm mass (Case 2)

Based on the large symptomatic mass and the uncertain nature of the lesion, an exploratory laparotomy was undertaken. Intraoperatively, the mass was identified as arising intramurally from the uterine fundus, without breaching the endometrial cavity. The fallopian tubes and ovaries bilaterally appeared grossly normal. The bulky tumor was well-encapsulated and extensively vascularized, occupying a significant portion of the abdominal cavity (Figure 7). Careful dissection allowed for surgical excision of the mass from its uterine attachments (Figure 8), and gross measurement confirmed a maximum dimension of approximately 30 cm (Figure 9).

Histopathological examination revealed a benign leiomyoma (fibroid) with no atypical mitotic activity or necrosis, and immunohistochemistry was positive for estrogen and progesterone receptors. The patient had an uneventful postoperative recovery and was discharged on postoperative day five. At six months follow-up, she remained free of symptoms, with no imaging evidence of recurrence. This case is particularly instructive as it emphasizes the challenges in diagnosing large pelvic masses in adolescents. The integration of clinical evaluation, laboratory data, and multimodal imaging was crucial in narrowing the differential diagnosis and avoiding overtreatment. The successful fertility-sparing surgical management further highlights the importance of a multidisciplinary approach in managing rare benign tumors in this age group.

## Discussion

Abdominal tumors in the pediatric and adolescent population constitute a heterogeneous group of pathologies with a broad range of clinical manifestations [1]. Ovarian teratomas and uterine leiomyomas, common in adult populations, are rare in adolescents and pose significant diagnostic and therapeutic challenges when they occur [7]. A critical appraisal of the literature reveals that many studies on these conditions are limited by small sample sizes and retrospective designs, with few systematic reviews available. Moreover, conflicting reports exist regarding the incidence of ovarian teratoma-associated anti-NMDAR encephalitis in younger populations, with some studies suggesting an increase in reported cases while others maintain that the condition remains rare. This variability underscores the need for larger, prospective studies to better define these epidemiological trends.

Ovarian teratoma-related anti-NMDAR encephalitis

The first case illustrates the complex interplay between a benign ovarian teratoma and anti-NMDAR encephalitis, a condition characterized by an autoimmune response against N-methyl-D-aspartate receptors in the brain [3]. Mechanistically, the presence of ectopic neural tissue within teratomas is believed to expose NMDA receptor antigens, which may trigger an autoimmune response via molecular mimicry. However, the exact immune triggers remain incompletely understood, and factors such as genetic predisposition may also contribute. Further research is needed to clarify these immunopathogenic mechanisms. Clinically, patients typically present with behavioral changes, psychosis, dyskinesias, seizures, and altered levels of consciousness [6]. Current treatment protocols generally involve first-line immunotherapies, such as corticosteroids, intravenous immunoglobulin (IVIG), and plasmapheresis, with second-line agents like rituximab reserved for refractory cases. In our case, despite a negative initial ultrasound, a high-resolution MRI identified a small teratoma, underscoring the importance of repeated and advanced imaging in cases with high clinical suspicion [11]. Furthermore, the normal levels of tumor markers such as α-fetoprotein and β-human chorionic gonadotropin highlight the limitations of these assays in diagnosing teratomas [12].

Uterine leiomyoma in adolescence

The second case pertains to a uterine leiomyoma (fibroid) presenting as a massive abdominal mass with progressive distension and anemia. Uterine fibroids are benign smooth muscle tumors of the uterus, predominantly occurring during reproductive years, with an incidence rising to 70%-80% in women by age 50 [9]. In contrast, leiomyomas in adolescents are exceedingly rare, with published data estimating an incidence of under 1% in this demographic [1]. When fibroids do manifest in younger patients, they often attain larger sizes and can present atypically, mimicking malignant processes [13]. Emerging evidence suggests that, in addition to hormonal influences, genetic mutations, particularly in the MED12 gene, may also contribute to the development of leiomyomas, although this has not been extensively studied in the adolescent population [14]. The significant mass effect, compressing adjacent structures such as the bladder and inferior vena cava, is not uncommon in fibroids exceeding 10 cm in diameter [8]. The imaging modalities employed, ultrasound, computed tomography, and magnetic resonance imaging, are pivotal for accurate lesion localization and surgical planning, with MRI often regarded as the gold standard due to its superior soft-tissue contrast resolution [8,15].

Management strategies for uterine fibroids in pediatric and adolescent populations generally mirror those used in adults but must also consider future fertility and growth potential [16]. Medical therapy (e.g., gonadotropin-releasing hormone agonists) is sometimes effective for symptom control but often fails to induce significant tumor size reduction [9]. Thus, surgical intervention, ranging from myomectomy to, in extreme cases, hysterectomy, may be necessary. In younger patients, myomectomy is preferred to preserve uterine function, and in our case, a laparotomy with tumor enucleation was performed. Histopathological confirmation of benign leiomyoma was essential to rule out malignancy, such as leiomyosarcoma, which can occasionally present with similar features [7].

Comparison with previously published reports

By situating our cases alongside previously documented reports, it becomes evident that both ovarian teratoma-related anti-NMDAR encephalitis and adolescent uterine leiomyomas can be diagnostically elusive. For example, Walker et al. reported a 14-year-old with severe anti-NMDAR encephalitis who underwent months of immunotherapy before a repeat imaging study uncovered an ovarian teratoma, initial imaging having been negative [17]. Their findings align closely with our Case 1, emphasizing that high clinical suspicion and repeated imaging are pivotal when no lesion is detected initially.

Similarly, Novi et al. described a case in which a large uterine leiomyoma caused acute urinary retention by compressing the bladder outlet [18]. Although their patient was older than our 14-year-old in Case 2, the parallel underscores how even benign fibroids can produce dramatic presentations due to mass effect. In our case, the bilobed leiomyoma, measuring up to 30 cm, caused significant bladder and bowel compression, reinforcing the principle that large tumors in adolescents may be mistaken for malignancies but can still be benign.

Our two cases contribute several novel insights to the existing literature. First, early multimodal evaluation is crucial in suspected anti-NMDAR encephalitis when initial imaging is inconclusive. Second, uterine fibroids in adolescence, though rare, can grow to substantial sizes and mimic malignant processes; thus, fertility-sparing surgical management demands meticulous planning. Future research might explore genetic or hormonal factors that predispose younger patients to these sizable tumors and also examine optimal imaging intervals for suspected paraneoplastic encephalitis. Additionally, these cases highlight the potential value of incorporating a more standardized approach to repeated imaging and fertility-preserving strategies in emerging clinical guidelines, aiming to detect occult pathology earlier and reduce morbidity.

Clinical implications and limitations

These two cases illustrate the diagnostic intricacies and therapeutic imperatives of abdominal tumors in adolescence. Ovarian teratoma-associated anti-NMDAR encephalitis requires high clinical suspicion for timely immunological and surgical management, an approach that can be lifesaving and disability-limiting [2,3]. Uterine leiomyomas, although benign, can grow to remarkable sizes and cause significant morbidity from mass effect if not recognized promptly [9]. A multidisciplinary collaboration involving pediatricians, gynecologists, radiologists, and neurologists is essential to optimize outcomes.

Despite the educational value and clinical relevance of these two case reports, several limitations persist. The small sample size inherent to single-institution, case-based research precludes broad generalizability. Long-term follow-up, beyond one year for Case 1 and six months for Case 2, is also lacking; further data could illuminate recurrence rates, fertility outcomes, and psychosocial impacts. Moreover, the immunological and molecular mechanisms that may predispose adolescents to these particular tumors remain poorly characterized. Larger, prospective studies examining these issues would provide a more robust evidence base for guiding imaging protocols, immunotherapy decisions, and surgical management in this population.

## Conclusions

Taken together, these two cases underscore the importance of recognizing atypical presentations of abdominal tumors in adolescent patients. In the first instance, early detection and surgical resection of ovarian teratoma in the setting of anti-NMDAR encephalitis proved vital for achieving prompt neurological recovery. In the second case, the identification and removal of a large uterine leiomyoma established a definitive diagnosis and alleviated significant symptoms. Notably, these cases highlight the utility of repeated, high-resolution imaging for detecting occult pathology and illustrate the benefits of a fertility-sparing approach in managing benign tumors.

Both cases illustrate that even benign tumors can produce considerable morbidity and emphasize the crucial role of meticulous clinical assessment, detailed imaging, and histopathological confirmation. A multidisciplinary approach is essential for ensuring accurate diagnosis, guiding treatment, and preserving fertility whenever possible. The insights gained from these presentations may inform future clinical guidelines, particularly regarding imaging protocols and early intervention strategies. Continued investigations into the genetic, hormonal, and immunologic underpinnings of these pathologies will be pivotal for refining diagnostic algorithms and improving patient outcomes. As these tumors can present with a broad range of clinical features, ranging from subtle neurologic changes to dramatic abdominal distension, heightened clinical awareness, repeated imaging when indicated, and timely intervention are crucial to optimize outcomes.
